# Adiponectin Upregulates MiR-133a in Cardiac Hypertrophy through AMPK Activation and Reduced ERK1/2 Phosphorylation

**DOI:** 10.1371/journal.pone.0148482

**Published:** 2016-02-04

**Authors:** Ying Li, Xiaojun Cai, Yuqing Guan, Lei Wang, Shuya Wang, Yueyan Li, Ying Fu, Xiaoyuan Gao, Guohai Su

**Affiliations:** 1 Department of Cardiology, Jinan Central Hospital Affiliated to Shandong University, Jinan, China; 2 Central Laboratory, Jinan Central Hospital Affiliated to Shandong University, Jinan, China; 3 School of Medicine, Shandong University, Jinan, China; University of Western Ontario, CANADA

## Abstract

Adiponectin and miR-133a are key regulators in cardiac hypertrophy. However, whether APN has a potential effect on miR-133a remains unclear. In this study, we aimed to investigate whether APN could regulate miR-133a expression in Angiotensin II (Ang II) induced cardiac hypertrophy in vivo and in vitro. Lentiviral-mediated adiponectin treatment attenuated cardiac hypertrophy induced by Ang II infusion in male wistar rats as determined by reduced cell surface area and mRNA levels of atrial natriuretic peptide (ANF) and brain natriuretic peptide (BNP), also the reduced left ventricular end-diastolic posterior wall thickness (LVPWd) and end-diastolic interventricular septal thickness (IVSd). Meanwhile, APN elevated miR-133a level which was downregulated by Ang II. To further investigate the underlying molecular mechanisms, we treated neonatal rat ventricular myocytes (NRVMs) with recombinant rat APN before Ang II stimulation. Pretreating cells with recombinant APN promoted AMP-activated protein kinase (AMPK) phosphorylation and inhibited ERK activation. By using the inhibitor of AMPK or a lentiviral vector expressing AMPK short hairpin RNA (shRNA) cancelled the positive effect of APN on miR-133a. The ERK inhibitor PD98059 reversed the downregulation of miR-133a induced by Ang II. These results indicated that the AMPK activation and ERK inhibition were responsible for the positive effect of APN on miR-133a. Furthermore, adiponectin receptor 1 (AdipoR1) mRNA expression was inhibited by Ang II stimulation. The positive effects of APN on AMPK activation and miR-133a, and the inhibitory effect on ERK phosphorylation were inhibited in NRVMs transfected with lentiviral AdipoR1shRNA. In addition, APN depressed the elevated expression of connective tissue growth factor (CTGF), a direct target of miR-133a, through the AMPK pathway. Taken together, our data indicated that APN reversed miR-133a levels through AMPK activation, reduced ERK1/2 phosphorylation in cardiomyocytes stimulated with Ang II, revealing a previously undemonstrated and important link between APN and miR-133a.

## Introduction

Cardiomyocyte hypertrophy is a maladaptive response to cardiac insults, such as hypertension, myocardial infarction, valvular heart disease, cardiomyopathy, and congenital heart disease. Initially, cardiomyocyte hypertrophy may serve as a compensatory response. However, prolonged cardiac hypertrophy leads to LV dilation, contractile dysfunction, and subsequent heart failure. Identifying underlying mechanisms for pathological cardiac hypertrophy is critically important for developing new strategies to protect against heart failure. Gaining a greater understanding of the mechanisms responsible for left ventricular hypertrophy, including intercellular crosstalk of multiple factors implicated in this process, may suggest novel therapeutic strategies.

MiRNAs are short noncoding single-stranded RNAs approximately 20 nt in length that have emerged as key post-transcriptional regulators of gene expression. By base pairing to the 3’ untranslated region (UTR) of their target mRNAs, miRNAs mediate mRNA degradation or translational repression. MiRNAs are predicted to regulate approximately one-third of the genome, and thus are potent mediators of cellular signaling [1]. MiRNAs have been implicated in diverse biological processes including cell proliferation, tissue morphogenesis, apoptosis, autophagy, tumorigenesis, and heart disease [2–4]. In the human heart, miR-133a is the most abundant miRNA and is involved in the regulation of cardiac hypertrophy and failure [5]. According to TargetScan prediction results, there are 400–500 putative mRNA targets for miR-133a. Numerous functional roles have been proposed, including regulating myoblast proliferation and differentiation[6], suppressing embryonic cardiomyocyte proliferation[7], preventing genetic cardiac hypertrophy[8], inhibiting cancer[9] and downregulating connective tissue growth factor[10]. The disease-associated profiles of miR-133a expression could be generated in response to hypertrophic stimuli elicited by variations in the activity of intracellular signaling pathways.

APN is a cytokine produced predominantly in adipose tissue, which exerts a protective role against cardiovascular pathology. It has been reported that APN could ameliorate hypertension, diabetes, dyslipidemia, coronary artery diseases, atherosclerosis and cardiac hypertrophy[11–15]. APN ameliorate such disorders by exerting anti-inflammatory, superoxide-suppressing, anti-hypertrophic effects in cardiomyocytes[16, 17]. It was determined that APN protected against Ang II induced cardiac fibrosis, possibly through AMP-activated protein kinase activation [18]. Although several studies have demonstrated that APN inhibits cardiac hypertrophy[15, 19–22], whether APN has an effect on miR-133a expression is unknown.

Connective tissue growth factor (CTGF) is a heparin-binding 38 kDa member of the CCN family. CTGF is involved in a wide range of biological activities including cell proliferation, angiogenesis, cell migration, extracellular matrix (ECM) production, fibrosis and apoptosis in different organs[23, 24]. In the heart, CTGF is an important mediator of fibrosis[25]. Increased CTGF expression was associated with fibroproliferative disorders[26], and CTGF inhibition or knockdown can inhibit the progression of fibrotic lesions[27, 28]. In Esther E. Creemers’s study, the 3’-UTR of CTGF was proved to be a direct target of miR-133a[10]. APN suppresses cardiac fibrosis. However, whether APN regulates CTGF in the heart is unclear.

Here, we hypothesized that APN may affect miR-133a expression in Ang II induced cardiac hypertrophy. Our results showed that APN reversed miR-133a expression level downregulated by Ang II in vivo and in vitro. AMPK activation and reduced ERK1/2 phosphorylation were responsible for APN positive regulation on miR-133a. These results provide new evidence for the mechanism underlying cardiac hypertrophy and may provide important insight into regulatory networks of miR-133a, revealing a previously undemonstrated and important link between APN and miR-133a.

## Materials and Methods

### Reagents and chemicals

DMEM and bovine calf serum were purchased from Gibco Co. (Carlsbad, CA, USA). Compound c, Ang II and PD98059 were obtained from Sigma-Aldrich (St Louis, MO, USA). Recombinant rat adiponectin was purchased from Biovision Co. (Palo Alto, USA).

### Experimental Animals

For lentiviral vector-mediated gene transfer in rat, wild-type male Wistar rats were treated with either a lentiviral vector expressing APN (2 × 10^8^ TU) or with a negative control virus delivered through the jugular vein 3 d before infusion with Ang II. Ang II infusion was performed using subcutaneously implanted osmotic mini-pumps (200 ng/kg/min) for four weeks as described[29]. The animals were divided into non-treated or Ang II treated groups. The control animals were implanted with sterile saline pumps. On days 7, 14, 28, rats were anesthetized with an intraperitoneal injection of 3% pentobarbital sodium (70 mg/kg). Prior to sacrificing, blood samples were collected into tubes containing potassium EDTA and centrifuged at 2,500 g for 10 min at 4°C to separate the plasma. The heart was removed immediately following euthanasia and rinsed with 0.9% saline (4°C) and collected for further study.

### Echocardiography of rat

The rats were anesthetized lightly using sodium pentobarbital (70mg/kg). The animals were imaged in the left lateral decubitus position using a Visual Sonics Vevo 770 machine equipped with a 30 MHz high frequency transducer. Images were captured from M-mode, two-dimensional (2-D), pulsewave (PW) Doppler. All measurements of nuclear magnetic resonance were calculated by the same observer based on the average of six consecutive cardiac cycles[30].

### Hematoxylin and eosin (H&E) staining and determination of cell surface area

Myocardial tissues were cut in a cross-section, fixed in 4% paraformaldehyde solution, and embedded in paraffin for tissue sections. Hematoxylin and eosin (H&E) staining was performed to facilitate quantification of cardiomyocyte. Images were captured using a bright field microscope (Olympus BX53 microscope) and were then analyzed for cell size using Image Pro Plus 7.0 software. The data shown represent analyses from three independent experiments. The surface area of cells from each group (100–200 cells / group) was determined and compared with the control group.

### Analysis of mRNA and miRNA expression by real-time PCR

Total RNA was extracted using TRIzol reagent (Invitrogen) according to the manufacturer’s specifications. cDNA was synthesized from 1 μg of RNA with a PrimeScript RT reagent kit with gDNA Eraser (Takara). Real-time PCR amplification reactions were performed with SYBR Premix Ex Taq kit with ROX (Takara) in triplicate using the ABI Prism 7900 Real-Time PCR machine. Gene expression was measured by the ΔΔCT method and was normalized to β-actin mRNA levels. The data are presented as the fold change in the expression of the gene of interest relative to the control groups. The primer sequences used were as follows: atrial natriuretic peptide (ANF) forward, 5-GGGGGTAGGATTGACAGGAT-3 and reverse, 5-GGATCTTTTGCGATCTGCTC-3; and brain natriuretic peptide (BNP) forward, 5- GCTGCTTTGGGCAGAAGATA -3’ and reverse, 5- GGAGTCTGCAGCCAGGAGGT -3; β-actin forward, 5- CGTTGACATCCGTAAAGACC -3 and reverse, 5- TAGAGCCACCAATCCACACA -3. For the miR-133a real-time PCR, we used a miRCURY LNA™ Universal RT microRNA PCR kit (Exiqon). Template RNA was adjusted to 5 ng/μl. cDNA synthesis was performed according to the manufacture’s instruction. The miR-133a and U6 expressions were evaluated by real-time PCR using the ABI PRISM 7900 Sequence Detection System. The miR-133a expression level was normalized to U6 expression following the ΔΔCT method. All real-time experiments have been repeated four times.

### Primary culture of neonatal rat ventricular myocytes (NRVMs)

NRVMs were prepared as previously described[31]. Briefly, 1- to 3-day-old Wistar rats were anesthetized with isoflurane and ventricles were minced and digested in phosphate buffered saline (PBS) containing 200 U type II collagenase and 0.4% horse serum for three cycles. The cells were then centrifuged and suspended in Dulbecco’s modified Eagle’s medium containing 5% fetal bovine serum and 8% horse serum. A single 1.5 h preplating step was used to further remove non-cardiomyocytes. Non-cardiomyocytes attached readily to the bottom of culture dishes. The unattached myocytes were plated at 1×10^5^ cells/ml in the same medium as above and supplemented with 0.1 mM 5-Bromo-2-deoxyUridine (BrdU). Cells were placed in a serum-free medium for 24 h before experiments. NRVM identity was confirmed by morphological examination and by staining with an anti-sarcomerica-actin antibody. Most (>95%) of the cells were identified as NRVMs.

### [3H]-leucine incorporation assay

NRVMs were seeded into 24-well plates at a density of 5×105 cells/ml, with 0.5 ml in each well. NRVMs cultured in24-well plates were serum-deprived for 24 h and then incubated with AngII or vehicle and [3H]-leucine (3.7×104 Bq/ml) for 48 h. Plates were then placed on ice, quickly washed three times with 1 ml ice-cold phosphate-buffered saline (PBS), incubated for 10 min with 1 ml trichloroacetic acid, and washed with 1 ml absolute methanol. Precipitates were solubilized for 30 min in 0.5 ml of 0.3 M NaOH–1% SDS at room temperature. The cell lysates were harvested, transferred to glass-fiber filter paper, and subjected to drying at 42°C. The radioactivity (cpm/cell) of the cells was measured by the use of a liquid scintillation counter (LS6500, Beckman Coulter, Brea, CA, USA).

### Lentiviral infection of NRVMs

ShRNAs targeting rattus norvegicus AMPKα2 catalytic subunit (5’- GCTGACTTCGGACTCTCTA -3’) and AdipoR1 (5’ -CGTCTACTGTTCAGAGAA-3’) were synthesized and cloned into pLKO.1-puro (Sigma-Aldrich, USA) to generate the lentiviral expression vectors, which were then transfected into 293 T cells with packaging plasmids pCMV-VSV-G and pCMV-dR8.2. Viral supernatant was harvested 48 h after transfection and the titer was detected. ShRNAs targeting 5’- TTCTCCGAACGTGTCACGT -3’) served as negative control. NRVMs were infected with recombinant adenovirus at the indicated multiplicity of infection and incubated for 72 h before experiments.

### Western blot analysis

Western blotting and quantification of the abundance of relative proteins were performed as described previously[32]. Briefly, cells were lysed in protein lysis buffer (1% SDS, 25 mM Tris–HCl (pH 7.5), 4 mM EDTA, 100 mM NaCl, 1 mM PMSF, 10 mg/ml leupeptin and 10 mg/ml soybean trypsin inhibitor). The protein concentration of the lysates was determined using the Coomassie Brilliant Blue protein assay. NRVM protein extracts (20 μg) were loaded on 12% SDS polyacrylamide gels, subjected to electrophoresis, and transferred to a nitrocellulose membrane. The membranes were incubated with anti-CTGF, anti-pAMPK (Thr 172), anti-AMPK, anti-ERK, anti-pERK (Thr 202 / Tyr 204), (Cell Signaling) or anti-β-actin antibodies (Santa Cruz Biotechnology) (1:1,000 dilution). The indicated proteins were detected with a horseradish peroxidase-conjugated IgG. The band intensity was quantified using Quantity One software (Bio-Rad, USA) and normalized to β-actin levels.

### Paraffin section immunohistochemistry

Samples were fixed in 4% formaldehyde in PBS (pH 7.2) and, after dehydration, embedded in paraffin wax and processed for immunohistochemical analysis of CTGF. Sections (5 mm thick) were cut and subsequently hydrated. Slides were incubated in hydrogen peroxide (3%) for 15 min at room temperature to quench the activity of endogenous peroxidase and then blocked with normal goat serum and anti-rat CTGF antibody (GeneTex, 1:500 diluted). Immunoreactivity was visualized using diaminobenzidine (DAB), a peroxidase substrate. The negative control omitted the primary antibody.

### Statistical Analysis

The data are presented as the mean ± SD. The statistical analysis of differences between two groups was assessed with the unpaired t—test, and the differences among more than three groups were assessed by one-way analysis of variation (ANOVA) followed by a Bonferroni’s tests for post hoc analysis and multiple comparison tests with Prism Software version 5.0 (GraphPad Software, San Diego California USA). Data presented in S1 File were analyzed by two-way repeated-measures ANOVA. The figures were processed with Adobe Photoshop software. The mean values were derived from at least three independent experiments. Differences with a *p* < 0.05 were considered statistically significant.

## Results

### Lentiviral vector-mediated APN overexpression attenuates AngII-induced cardiac hypertrophy

To assess cardiomyocyte hypertrophy in vivo, we performed echocardiography to assess ventricular wall thickness, and determined left ventricle weight to body weight (LVM/body weight) and the expression of the specific markers ANF and BNP, on days 7, 14, 28 after Ang II infusion (200 ng/kg/min) in Wistar male rats. The results showed that Ang II infusion for 14 days promoted cardiac hypertrophy, and became much more significant for 28 days as shown in S1 File. Ang II infusion (for 28 days) induced a significant increase in LVPWd by 27.92% and IVSd by 28.12% compared to control group (Fig A and Fig B in S1 File). In parallel with the echocardiography data, LVM/body weight was significantly increased by 16.53% in Ang II infusion for 28 days group compared to the control group (Fig C in S1 File). Ang II infusion also elevated the mRNA expression of the specific markers ANF (by 162.83%) and BNP (by 174.34%) after 28 days compared to the control group (Fig D and Fig E in S1 File). In addition, Plasma adiponectin level was significantly decreased by 36.71% (28 day) compared to the control groups (Fig F in S1 File).

**Fig 1:**
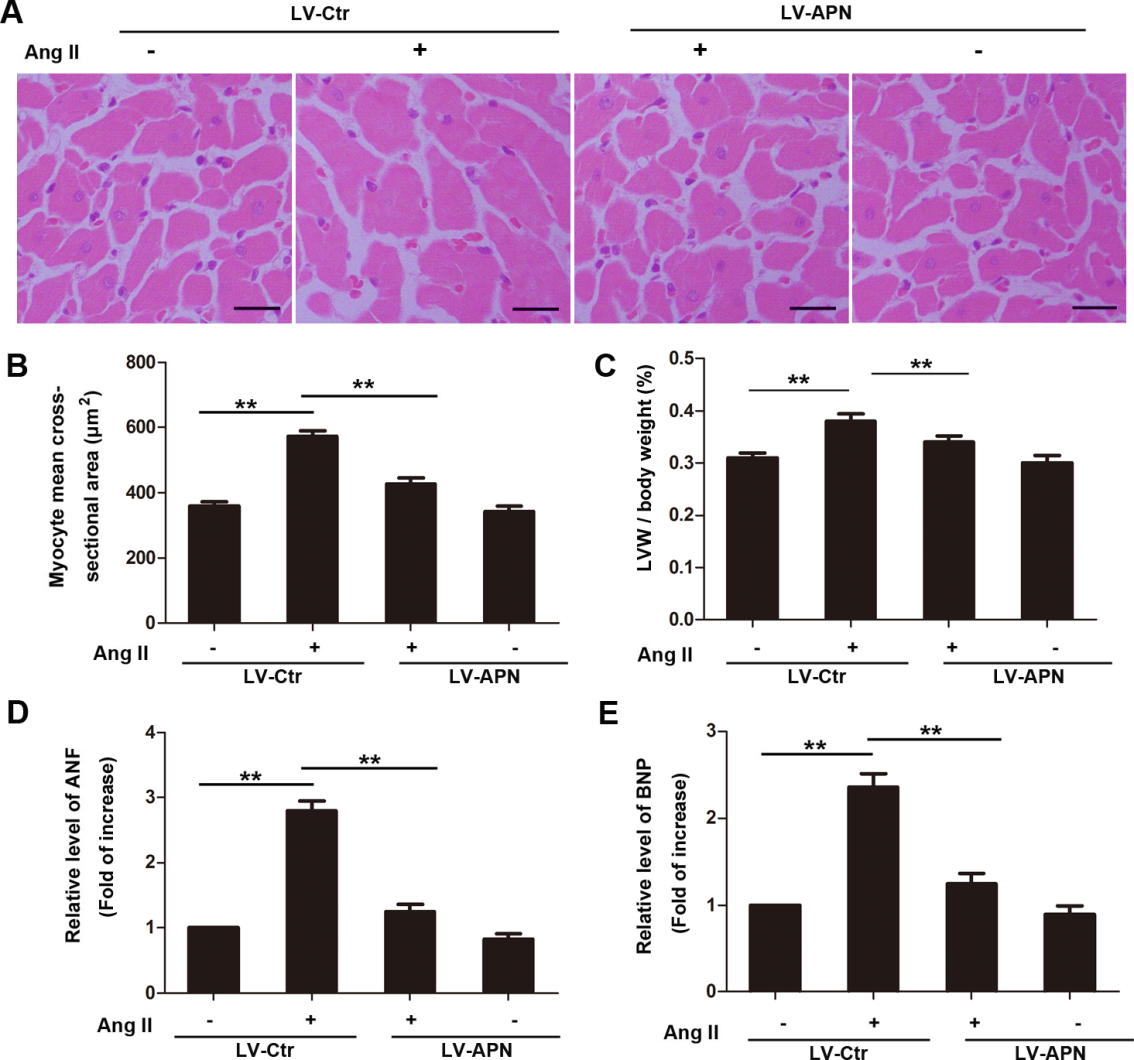
Lentiviral overproduction of APN inhibited Ang II-induced cardiac hypertrophy. For lentiviral vector-mediated gene transfer, wild-type Wistar rats were treated with a lentiviral vector expressing APN (LV-APN) or with a control virus (LV-Ctr) delivered through the jugular vein 3 d before infusion with Ang II. Ang II was infused (200 ng/kg/min) for 28 days into those rats. (A) Cross-sectional area of the cardiomyocytes in rats was assessed by hematoxylin and eosin (H&E) staining. Bar represents 20 μm. (B) Quantification of cell size and (C) heart weight / body weight ratio. Total RNA was isolated from the left ventricles of rats, and subjected to real-time RT-PCR for ANF (D) and BNP (E). Amplification curves were normalized to β-actin. All samples were analyzed in triplicate and expressed as the mean ± SD. (**, *p*< 0.01, n = 6 for each group).

**Fig 2:**
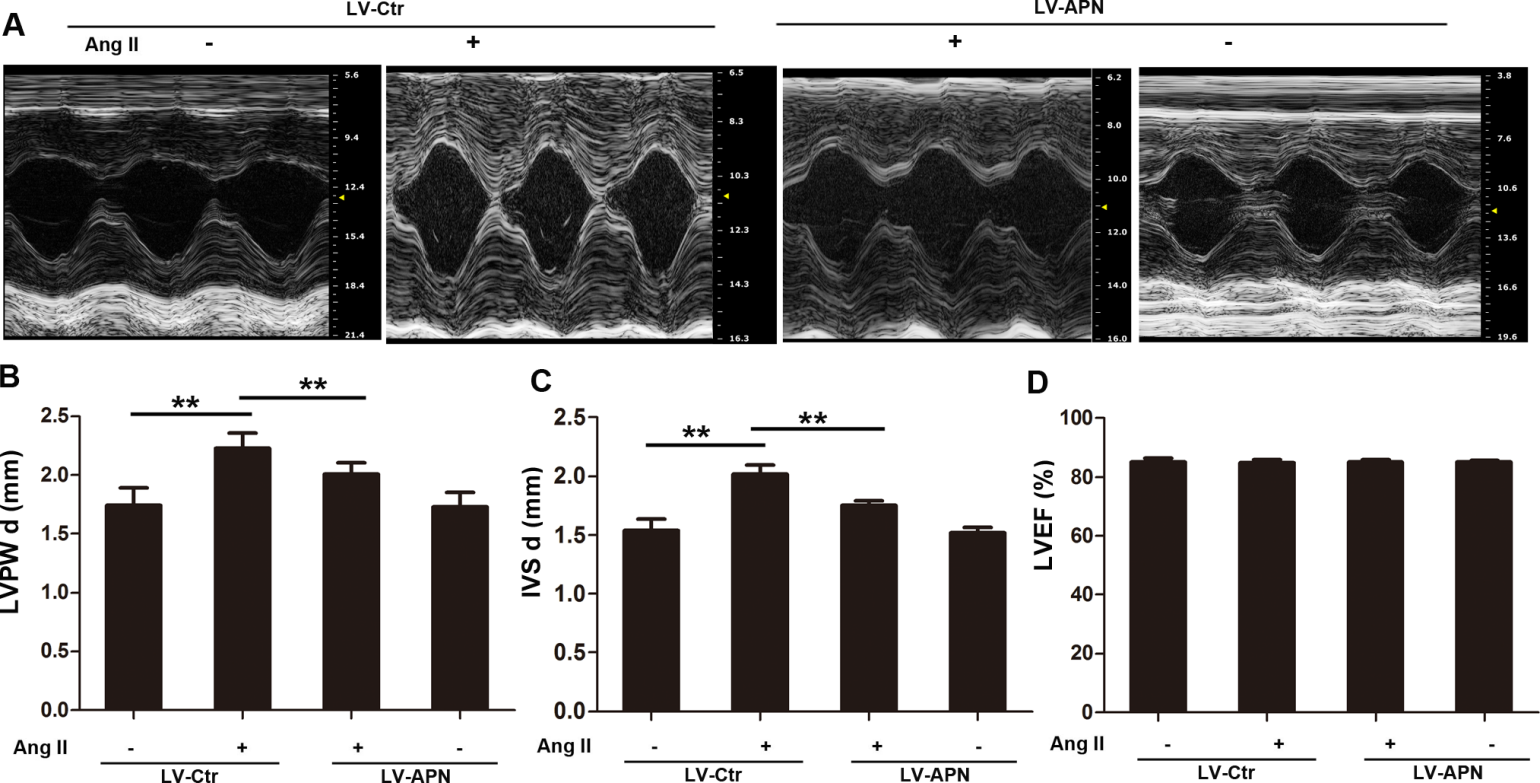
Echcardiography showed that lentiviral vector-mediated APN overexpression improve Ang II stimulated hypertrophy. (A) M-mode tracings. Representative photographs were shown. The left ventricular end-diastolic posterior wall thickness (LVPWd) and end-diastolic interventricular septal thickness (IVSd) were shown in (B) and (C). (D), ejection fraction of left ventricle (LVEF). Data represent mean ± SD. (**, *p* < 0.01. n = 6).

To detect the effect of APN on cardiac hypertrophy induced by Ang II infusion, wild-type male Wistar rats were treated with either a lentiviral vector expressing APN (2 × 10^8^ TU) or with a negative control virus delivered through the jugular vein 3 d before Ang II treatment. Lentiviral vector-mediated APN (LV-APN) treatment increased APN level significantly in heart (S2 File) and ameliorated those Ang II–induced cardiac hypertrophy responses as shown in Fig 1. Ang II infusion for 28 days induced larger cell surface area (359.2±13.098 μm^2^ in control rats versus 573.13±17.025 μm^2^ in Ang II group). However, the increase was inhibited by 68.89% in LV-APN group (Fig 1A and 1B). Meanwhile, the increase of LVM/body weight, mRNA expression of ANF and BNP were inhibited by 57.14%, 86.03%, 82.22% respectively in LV-APN group (Fig 1C–1E). In addition, the protective role of APN in Ang II induced hypertrophy was also confirmed by echocardiography (Fig 2A). The increase of LVPWd and IVSd induced by ang II infusion were decreased by 44.9% and 58.82% respectively in LV-APN group (Fig 2B and 2C). The left ventricular ejection fraction (LVEF) was not affected (Fig 2D). These results demonstrated that lentiviral-mediated APN supplemention attenuated the Ang II–induced cardiac hypertrophy responses.

### APN reversed miR-133a downregulation by Ang II

**Fig 3:**
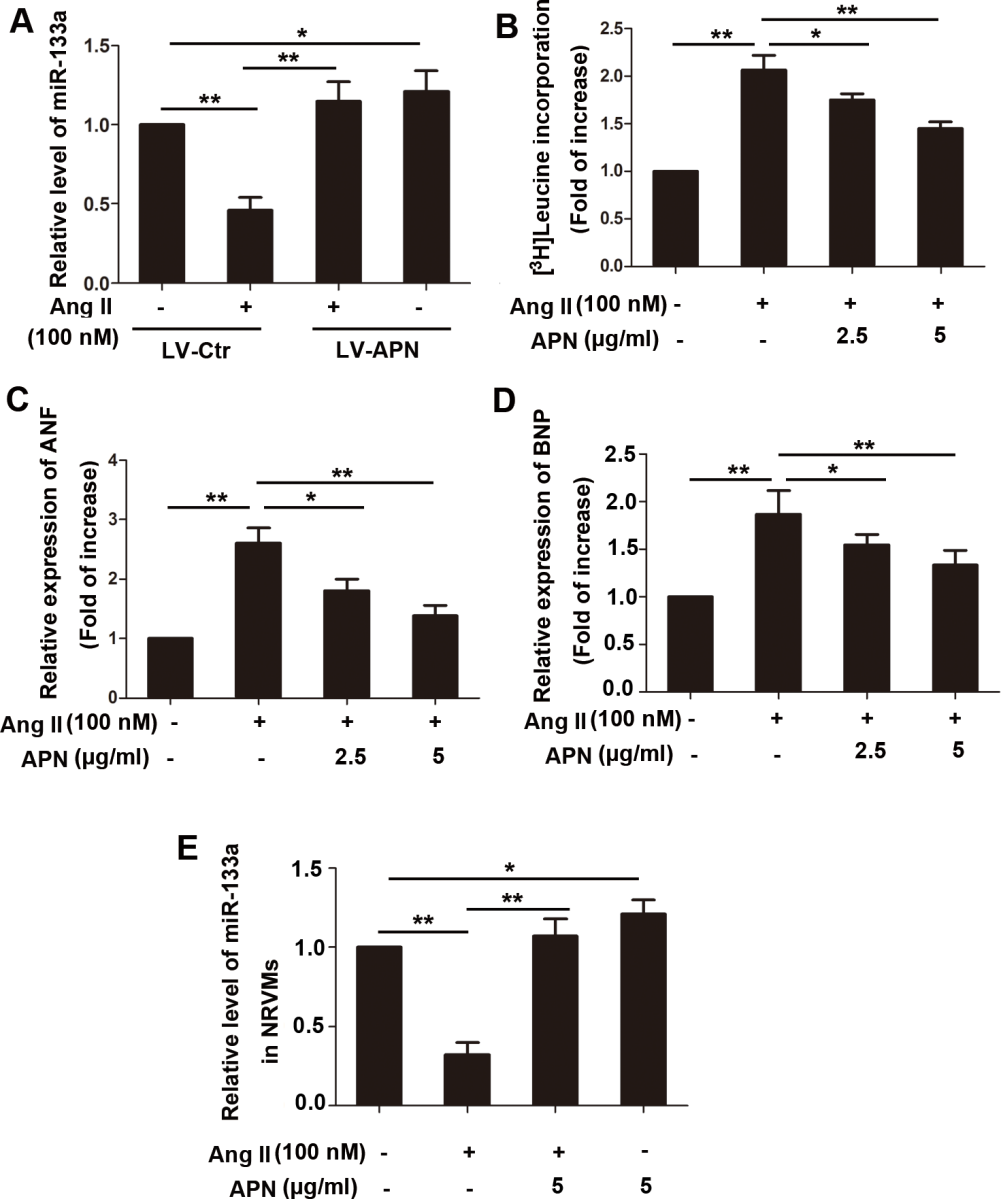
APN attenuated cardiac hypertrophy in vitro and reversed miR-133a downregulation by Ang II in vivo and in vitro. (A) Lentiviral—mediated APN overexpression and supplement of recombinant APN reversed miR-133a downregulation by Ang II in vivo. (B) NRVMs underwent 100 nM Ang II stimulation in the presence or absence of APN (2 or 5 μg/ml) with 1.0 mCi/ml [^3^H]-leucine for 24 h. APN was applied to NRVMs 60 min before Ang II stimulation. To remove the possibility that the increased protein synthesis is hyperplasia, the data were normalized to cell numbers. (C) Total RNA was isolated from the NRVMs and subjected to real-time RT-PCR for ANF and (D) BNP, and (E) miR-133a. Amplification curves were normalized to β-actin or U6 snRNA (**, *p* < 0.01. *, *p* < 0.05, n = 6).

MiR-133a is downregulated in cardiac hypertrophic responses. However, whether APN modulates miR-133a expression is unknown. Here, we show that MiR-133a was reduced by 54.12% in Ang II infused heart tissue compared with that in the control rats, whereas lentiviral overproduction of APN attenuated the miR-133a reduction by 69% (Fig 3A). In vitro, APN also alleviated cardiomyocyte hypertrophy as shwn in Fig 3B–3D. Ang II (100 nM) treatment for 24 h in NRVMs induced a 106.7% elevation in the cellular incorporation of [3H]-leucine, which were significantly decreased by 2.5 or 5μg/ml recombinant rat APN pretreatment for 2 h (Fig 3B). The expressions of ANP and BNP mRNA were significantly increased by 160% and 86% (*P* < 0.01) with Ang II stimulation, whereas recombinant rat APN pretreatment markedly reduced the increased mRNA expression (*P* < 0.01) (Fig 3C and 3D).

To determine the effect of Ang II on miR-133a expression, NRVMs were stimulated with 50, 100, 200, 500 nM Ang II for 24 h, or with 100 nM Ang II for 3, 6, 12, 24, 48 h. The results showed that miR-133a was repressed significantly in a dose and time-dependent manner in NRVMs stimulated Ang II (S3 File). Recombinant rat APN pretreatment (5 μg/ml) markedly attenuated the miR-133a reduction caused by Ang II (100 nM) stimulation for 24h (Fig 3E).

### APN reversed the miR-133a reduction in the Ang II mediated hypertrophic response via the AMPK pathway

**Fig 4:**
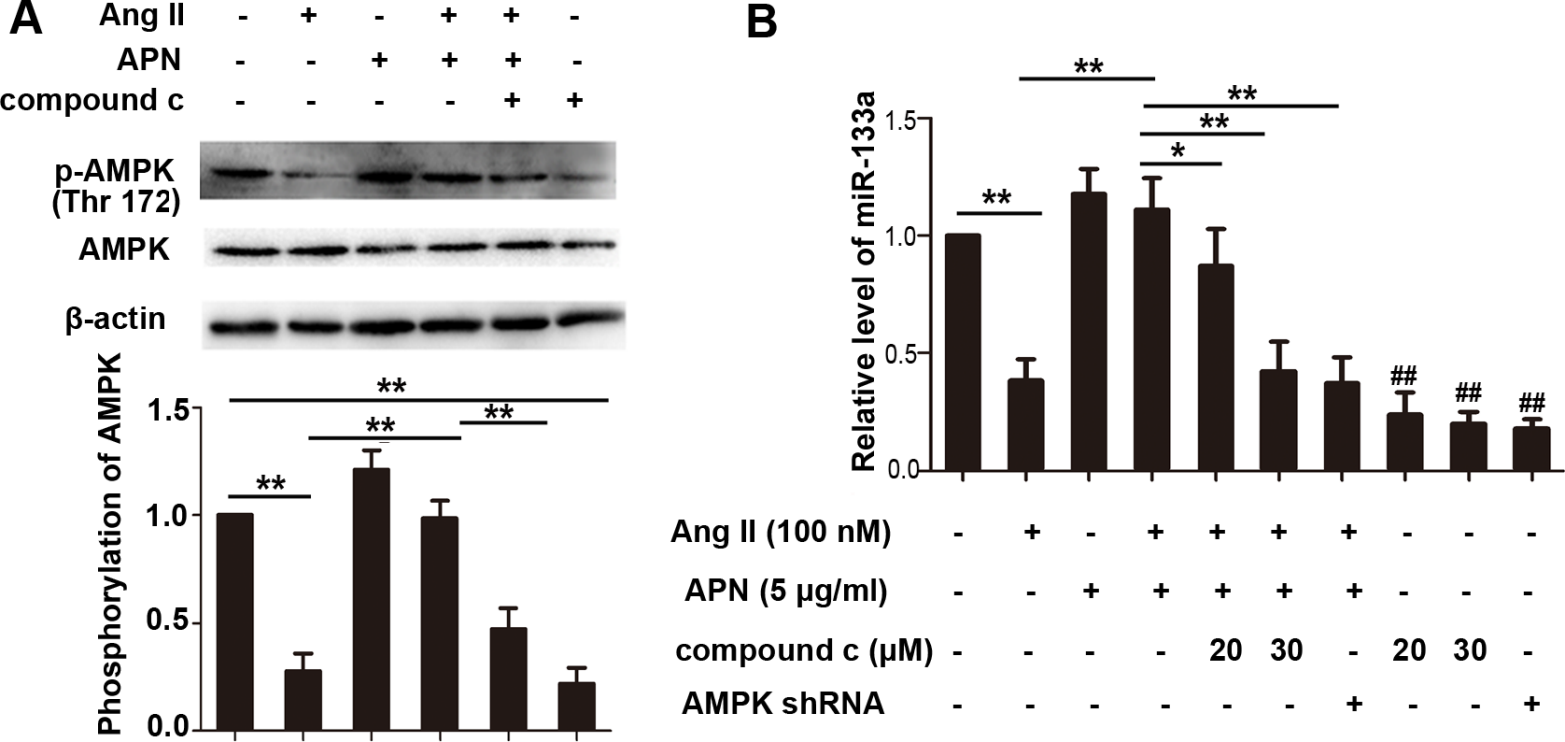
APN upregulated miR-133a through AMPK pathway in the Ang II mediated hypertrophic responses. (A) Phosphorylated (p-) AMPK and AMPK level were measured by Western blot in NRVMs. (B) miR-133a level was detected by real-time PCR. NRVMs were transduced with lentiviral AMPK shRNA or Scramble controls and then treated with the indicated regents. Amplification curves were normalized to U6 snRNA (**, *p*< 0.01. ##, *p* < 0.01 vs control group. n = 3).

AMPK plays a key role in adiponectin-mediated cardiovascular protection [33]. However, whether miR-133a upregulation by APN occurred through the AMPK pathway was not known. We next sought to examine whether the positive effect of APN on miR-133a was mediated by AMPK. As shown in Fig 4A, Ang II stimulation decreased AMPK phosphorylation, while incubation with recombinant APN significantly activated AMPK in NRVMs. Pretreatment of compound c (20 μM) for 3 h, which was an inhibitor of AMPK, antagonized the effect of APN on AMPK activation. To further test whether AMPK activation was responsible for miR-133a regulation by APN, we determined the miR-133a level in NRVMs following the treatments indicated in Fig 4B. The results demonstrated that as an activator of AMPK, APN increased miR-133a level which was suppressed by Ang II. The AMPK inhibitor compound c (20 μM) weakened the effects of APN, and 30 μM compound c canceled the effect of APN on miR-133a. To further confirm the effect of AMPK on miR-133a expression, a lentiviral vector expressing AMPK short hairpin RNA (shRNA) was constructed and infected into NRVMs. Interference efficiency was determined by real-time quantitative PCR (S4 File). NRVMs transduced with lentiviral AMPK shRNA canceled the effect of APN on miR-133a, indicating that APN may regulate miR-133a through the AMPK pathway (Fig 4). In addition, mir-133a also decreased significantly while using the AMPK inhibitor compound c or lentiviral AMPK shRNA alone, suggesting an important effect of AMPK pathway on miR-133a expression.

### APN upregulated miR-133a levels through inhibiting ERK1/2 phosphorylation

**Fig 5:**
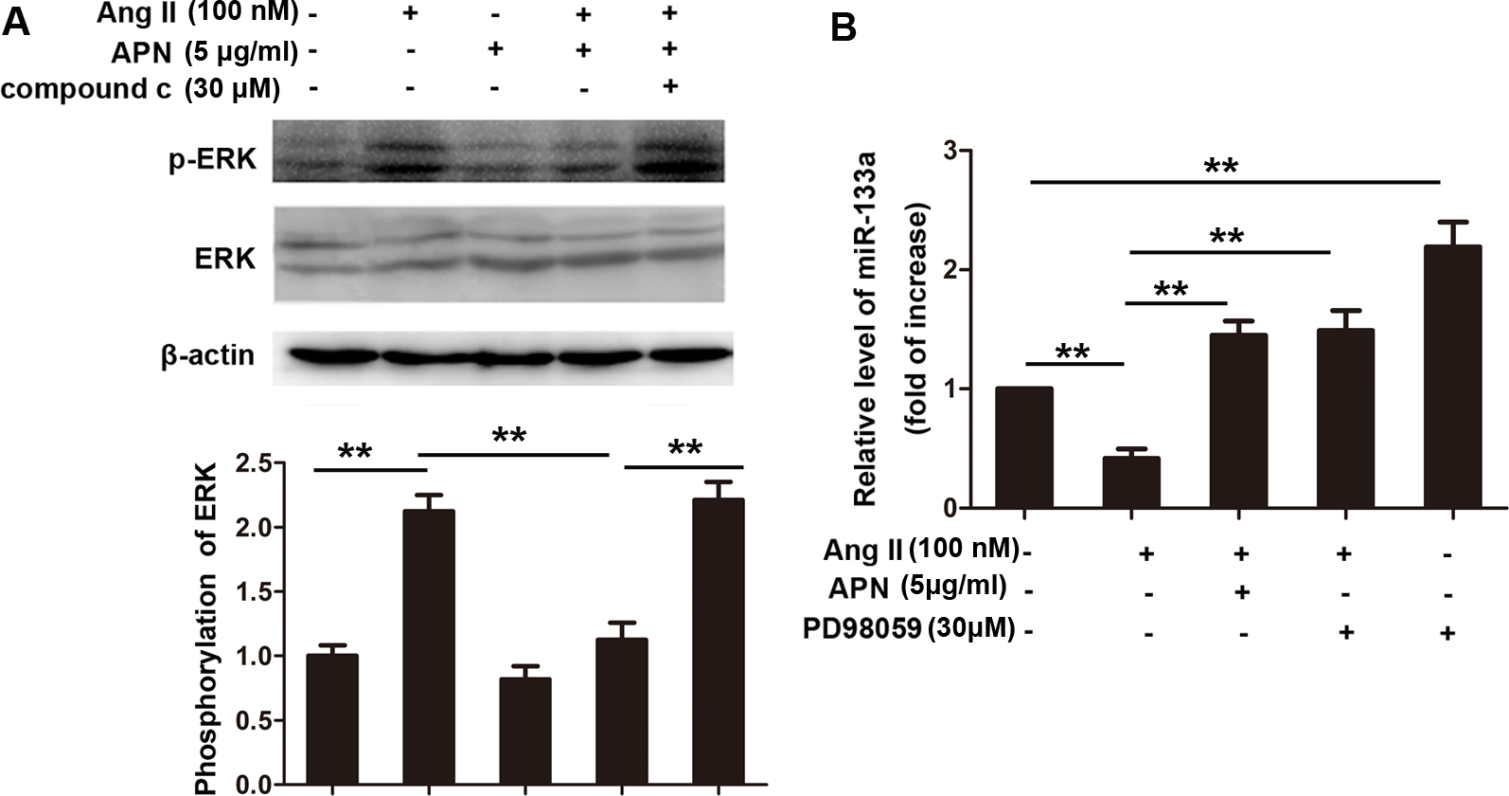
APN upregulated miR-133a levels by inhibiting ERK1/2 phosphorylation. (A) p-ERK and ERK expression level were determined by Western blot. (B) miR-133a level was detected by real-time RT-PCR. The miR-133a expression level was normalized to U6 expression following the ΔΔCT method. (**, *p*< 0.01, n = 3).

A large body of evidence has supported an important role of the ERK1/2 kinase in the development of cardiac hypertrophy [34–36]. However, whether ERK1/2 involved in APN mediated regulation of miR-133a in cardiac hypertrophy is unknown. Thus, we next detected whether APN prevented the downregulation of miR-133a through the ERK1/2 pathway. First, we determined ERK phosphorylation after stimulation with 100 nM Ang II in NRVMs for different time (S5 File). The results showed that Ang II stimulation increased the ERK phosphorylation levels significantly in NRVMs and the peak time was 12 h. AngII (100 nM) treatment for 10 min in NRVMs induced a 112.3% elevation in the ERK phosphorylation levels. APN (5 μg/ml) pretreatment for 2 h reversed these changes (Fig 5A). An AMPK inhibitor, compound c (30 μM), cancelled the effect of APN on the ERK phosphorylation. When pretreating NRVMs with the ERK1/2 inhibitor PD98059 (30 μM) for 1 hour before stimulation with Ang II, we observed that miR-133a expression increased significantly compared with the Ang II treatment group (Fig 5B). These results indicated that APN reversed the miR-133a downregulation induced by Ang II by ameliorating ERK1/2 phosphorylation. In addition, by using the ERK1/2 inhibitor PD98059 alone could also elevate the miR-133a level, suggesting that ERK1/2 may involve in the regulation of miR-133a in heart. We also detected the ERK phosphorylation level in LV-APN transfected rat with or without Ang II treatment, the results were consistent with that observed in vitro (S6 File).

### AdipoR1 was responsible in mediating the adiponectin signals

**Fig 6:**
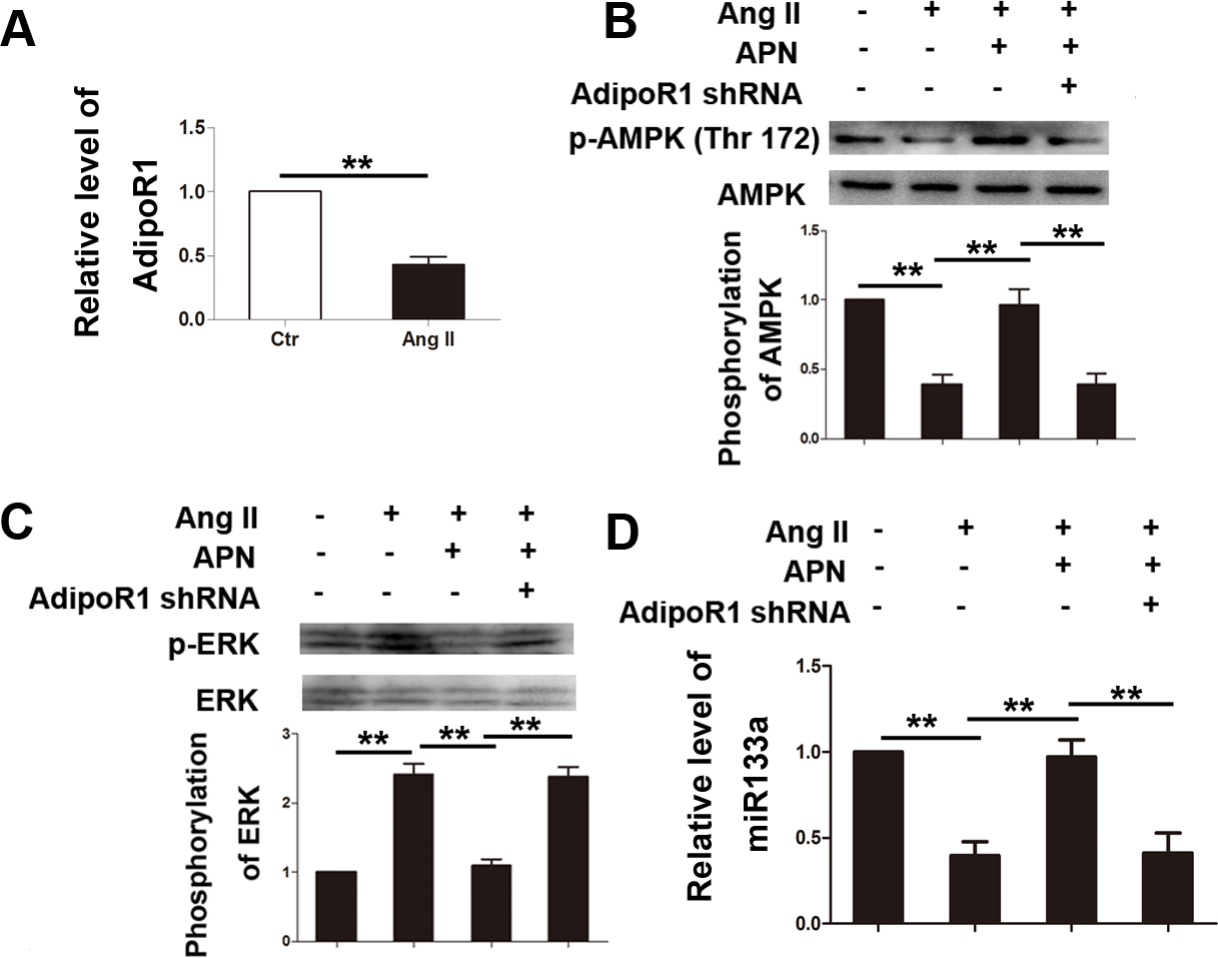
AdipoR1 was responsible in mediating APN signals. (A) AdipoR1 was inhibited by Ang II stimulation as determined by qPCR. Western blot was performed to determine phosphorylation of AMPK (B) and (C) ERK. (D) miR-133a level was determined by qRT-PCR (**, p < 0.01).

Two types of adiponectin receptors (AdipoRs), AdipoR1 and AdipoR2, mediate most effects of adiponectin [37]. To determine which receptor was responsible for the effect of APN on miR-133a in Ang II induced cardiac hypertrophy, we firstly detected AdipoR1 and AdipoR2 mRNA expression in NRVMs stimulated with Ang II. The results showed that the expression of AdipoR1 mRNA was significantly decreased by 57.33% under Ang II stimulation (Fig 6A). Expression of AdipoR2 had no change upon incubation with AngII (S7 File). To further investigate whether AdipoR1 was responsible in mediating APN signals, a lentiviral vector expressing AdipoR1 shRNA was constructed and infected into NRVMs. Interference efficiency was determined by real-time quantitative PCR (S8 File). The positive effect of APN on AMPK phosphorylation and miR-133a, and inhibitory effect on ERK phosphorylation was dramatically attenuated in NRVMs transfected with lentiviral AdipoR1 shRNA (Fig 6B–6D).

### APN suppressed increased CTGF expression caused by Ang II

**Fig 7:**
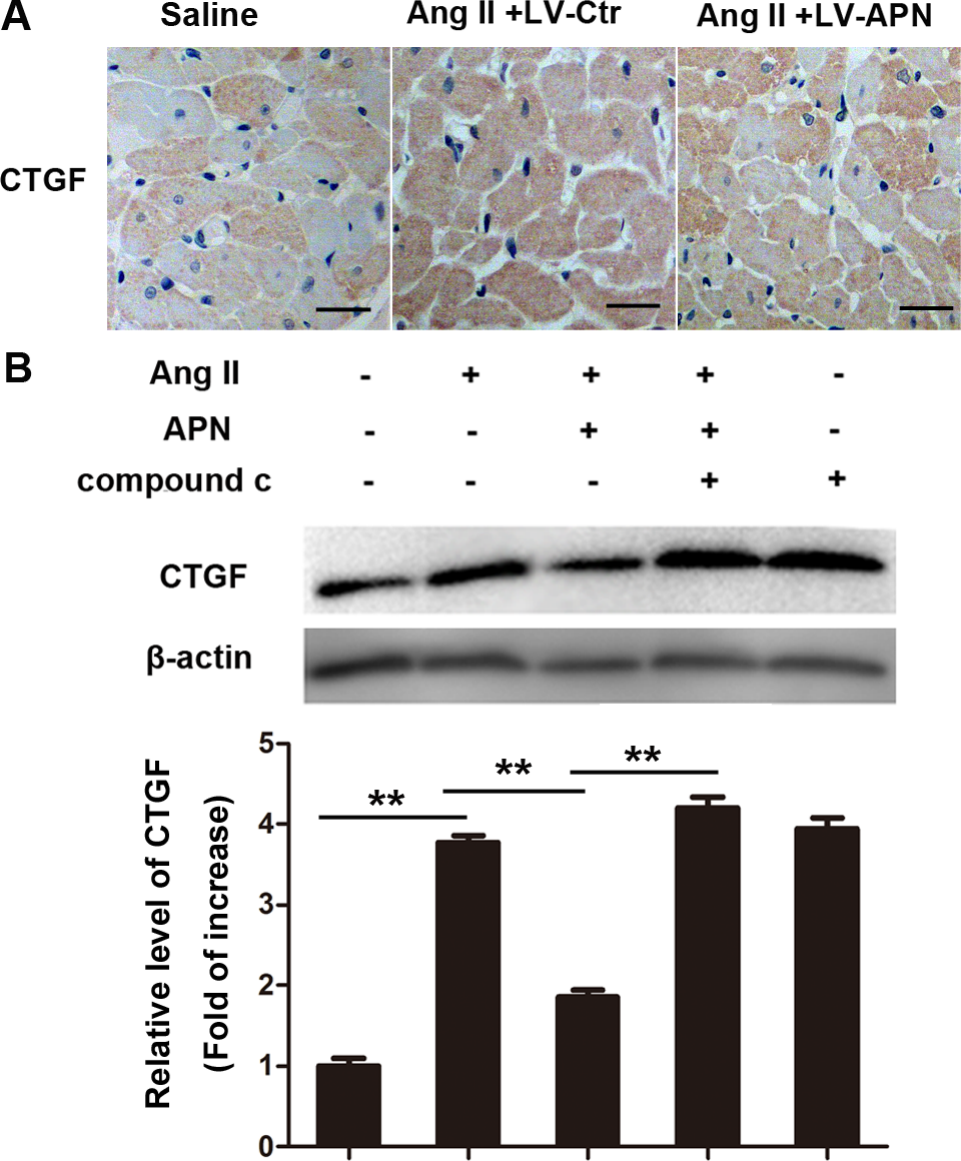
APN suppressed increased CTGF expression caused by Ang II. (A) CTGF levels were measured in the rat left ventricle by immunohistochemistry using CTGF antibodies. (B) CTGF expression in NRVMs were determined by Western blot (**, *p*< 0.01. n = 3).

CTGF is a direct target of miR-133a[10]. Thus, we determined whether APN could affect expression of CTGF. As displayed in Fig 7A, CTGF increased in the Ang II infused heart and was attenuated in the lentiviral-APN transfected heart. Treatment with 100 nM Ang II for 24 h significantly increased CTGF expression in NRVMs (Fig 7B). Pretreatment with APN (5 μg/ml) diminished the increase of CTGF. The AMPK inhibitor compound c cancelled the effect of APN. These results suggest that APN may inhibit CTGF expression through the AMPK pathway.

## Discussion

Recently, miRNAs have gained attention due to their key roles in physiological or pathological conditions. In previous studies, miR-133a was found to be specifically expressed in cardiac and skeletal muscle and proved to play a key role in skeletal and cardiac muscle development and function. Its aberrant expression has been linked to many diseases including cardiac hypertrophy[38], cardiac fibrosis[39], muscular dystrophy[40], heart failure[41], and cardiac arrhythmia[42, 43]. According to TargetScan prediction results, there are hundreds of mRNAs targeted by miR-133a. Thus, a network of genes can be subject to coordinated and simultaneous regulation by miR-133a. A change in the pattern of miRNAs expressed in a cardiomyocyte can generate genome-wide remodeling of gene expression and consequently induce hypertrophy[44]. According to the previous studies, a few factors have been identified involved in the regulation of miR-133a, such as serum response factor (SRF)[45], MEF2 [46] and IP3[42]. Here, we show for the first time that APN could attenuate the downregulation of miR-133a induced by Ang II through AMPK activation, reduced ERK1/2 phosphorylation, revealing a previously undemonstrated and important link between APN and miR-133a.

AMPK is a metabolite-sensing protein kinase, which represents the mammalian form of the core component of a kinase cascade. It has been determined that APN functions to induce AMPK signaling in many cell types, such as skeletal muscle, liver, adipocytes, endothelial cells and NRVMs[17, 47–50]. However, whether AMPK is involved in the regulation of miR-133a is unknown. Our results show that APN upregulates miR-133a through AMPK activation. By using compound c, which is a inhibitor of AMPK, cancelled the APN’s effect. These results indicated AMPK was an important mediator in the regulation of miR-133a.

In the present study, we show that APN reversed the downregulation of miR-133a stimulated by Ang II through the AMPK and ERK1/2 pathway in NRVMs. In S-H Zhao’s study, a new feedback loop between miR-133 and the ERK1/2 signaling pathway involving an exquisite mechanism for regulating myogenesis was revealed in C2C12 (Mouse myoblast cell line) cells [6]. In their study, downregulation of ERK1/2 phosphorylation by miR-133 was detected. Furthermore, miR-133 expression was also negatively regulated by the ERK1/2 signaling pathway. These results were consistent with our study in the heart.

CTGF was shown to be a direct target of miR-133a in the previous study[10]. Thus, a large number of CTGF may act as a miRNA “sponge” and modulate the derepression of miRNAs in turn. In our study, APN inhibited the increased CTGF expression induced by Ang II, which may be one reason for the modulation of miR-133a and the protection role of APN. According to these results, we speculate that APN may also affect other miR-133a target proteins in the heart, which need further investigation.

Adiponectin is a circulating adipose-derived cytokine that has been reported to exert an anti-hypertrophy effect in different cardiac hypertrophy models [15, 17]. MiR-133a also plays a protective role in cardiac hypertrophy. Knockdown of miR-133a was sufficient to induce cardiac hypertrophy[8]. However, transgenic expression of miR-133a inhibited myocardial fibrosis and improved diastolic function without affecting the extent of hypertrophy in pressure-overloaded adult hearts [51]. Thus, we speculate that APN plays a protective role in myocardial partly through its positive effect on miR-133a.

## Conclusions

In summary, this study shows for the first time that APN reverses miR-133a level which is downregulated by Ang II through AMPK activation, reduced ERK1/2 phosphorylation in cardiomyocytes, revealing a previously undemonstrated and important link between APN and miR-133a. These data may provide new evidence for the regulation of miR-133a.

## Supporting Information

S1 File Continuous Ang-II infusion results in cardiac hypertrophy. The left ventricular end-diastolic posterior wall thickness (LVPWd) **(Fig A)**, and end-diastolic interventricular septal thickness (IVSd) **(Fig B)** and Left ventricular weight index (LVW/BW) were increased induced by Ang II **(Fig C)**. ANF **(Fig D)** and **(Fig E)** BNP mRNA level was elevated by Ang II. The mRNA expression was calculated as fold induction compared to the control 7d group Plasma APN was decreased by Ang II infusion **(Fig F)**. (n = 6 for each group. *, *p* < 0.05 vs control. **, *p* < 0.01 vs control).
(DOCX)

S2 File lentiviral vector-mediated APN overexpression was determined by western blot. (**, *p* < 0.01, n = 6 for each group).
(DOCX)

S3 File Ang II downregulate miR-133a level in a dose and time- dependent manner. qRT-PCR was performed to detect miR-133a level under different treatment (**, *p* < 0.01 vs control).
(DOCX)

S4 File qPCR was performed to examine AMPK mRNA level after transfected with lentiviral AMPK shRNA. (**, *p* < 0.01).
(DOCX)

S5 File Western blot was performed to determine phosphorylation of ERK in NRVMs treated with Ang II for different time. (**, *p* < 0.01 vs control. *, *p* < 0.05 vs control).
(DOCX)

S6 File Western blot was performed to determine phosphorylation of ERK in different treatment in vivo. (**, *p* < 0.01).
(DOCX)

S7 File AdipoR2 mRNA level was not changed by stimulation with Ang II. (DOCX)

S8 File qPCR was performed to examine AdipoR1 mRNA level after transfected with lentiviral AdipoR1 shRNA. (**, *p* < 0.01).
(DOCX)

## References

[1] Lewis BP, Burge CB, Bartel DP. Conserved seed pairing, often flanked by adenosines, indicates that thousands of human genes are microRNA targets. Cell. 2005;120(1):15–20. 10.1016/j.cell.2004.12.035 . 15652477

[2] Ambros V. The functions of animal microRNAs. Nature. 2004;431(7006):350–5. 10.1038/nature02871 . 15372042

[3] Yin X, Peng C, Ning W, Li C, Ren Z, Zhang J, et al miR-30a downregulation aggravates pressure overload-induced cardiomyocyte hypertrophy. Molecular and cellular biochemistry. 2013;379(1–2):1–6. 10.1007/s11010-012-1552-z . 23660952

[4] Wang J, Yang X. The function of miRNA in cardiac hypertrophy. Cellular and molecular life sciences: CMLS. 2012;69(21):3561–70. 10.1007/s00018-012-1126-y 22926414 PMC3474911

[5] Luo X, Zhang H, Xiao J, Wang Z. Regulation of human cardiac ion channel genes by microRNAs: theoretical perspective and pathophysiological implications. Cellular physiology and biochemistry: international journal of experimental cellular physiology, biochemistry, and pharmacology. 2010;25(6):571–86. 10.1159/000315076 . 20511702

[6] Feng Y, Niu LL, Wei W, Zhang WY, Li XY, Cao JH, et al A feedback circuit between miR-133 and the ERK1/2 pathway involving an exquisite mechanism for regulating myoblast proliferation and differentiation. Cell death & disease. 2013;4:e934 10.1038/cddis.2013.462 24287695 PMC3847338

[7] Liu N, Bezprozvannaya S, Williams AH, Qi X, Richardson JA, Bassel-Duby R, et al microRNA-133a regulates cardiomyocyte proliferation and suppresses smooth muscle gene expression in the heart. Genes & development. 2008;22(23):3242–54. 10.1101/gad.1738708 19015276 PMC2600761

[8] Care A, Catalucci D, Felicetti F, Bonci D, Addario A, Gallo P, et al MicroRNA-133 controls cardiac hypertrophy. Nature medicine. 2007;13(5):613–8. 10.1038/nm1582 . 17468766

[9] Luo J, Zhou J, Cheng Q, Zhou C, Ding Z. Role of microRNA-133a in epithelial ovarian cancer pathogenesis and progression. Oncology letters. 2014;7(4):1043–8. 10.3892/ol.2014.1841 24944666 PMC3961467

[10] Duisters RF, Tijsen AJ, Schroen B, Leenders JJ, Lentink V, van der Made I, et al miR-133 and miR-30 regulate connective tissue growth factor: implications for a role of microRNAs in myocardial matrix remodeling. Circulation research. 2009;104(2):170–8, 6p following 8. 10.1161/CIRCRESAHA.108.182535 . 19096030

[11] Ouchi N, Ohishi M, Kihara S, Funahashi T, Nakamura T, Nagaretani H, et al Association of hypoadiponectinemia with impaired vasoreactivity. Hypertension. 2003;42(3):231–4. 10.1161/01.HYP.0000083488.67550.B8 . 12860835

[12] Chang J, Li Y, Huang Y, Lam KS, Hoo RL, Wong WT, et al Adiponectin prevents diabetic premature senescence of endothelial progenitor cells and promotes endothelial repair by suppressing the p38 MAP kinase/p16INK4A signaling pathway. Diabetes. 2010;59(11):2949–59. 10.2337/db10-0582 20802255 PMC2963556

[13] Okamoto Y. Adiponectin provides cardiovascular protection in metabolic syndrome. Cardiology research and practice. 2011;2011:313179 10.4061/2011/313179 21318102 PMC3034991

[14] Fang F, Liu GC, Kim C, Yassa R, Zhou J, Scholey JW. Adiponectin attenuates angiotensin II-induced oxidative stress in renal tubular cells through AMPK and cAMP-Epac signal transduction pathways. American journal of physiology Renal physiology. 2013;304(11):F1366–74. 10.1152/ajprenal.00137.2012 . 23535586

[15] Essick EE, Ouchi N, Wilson RM, Ohashi K, Ghobrial J, Shibata R, et al Adiponectin mediates cardioprotection in oxidative stress-induced cardiac myocyte remodeling. American journal of physiology Heart and circulatory physiology. 2011;301(3):H984–93. 10.1152/ajpheart.00428.2011 21666115 PMC3191107

[16] Ouchi N, Shibata R, Walsh K. Cardioprotection by adiponectin. Trends in cardiovascular medicine. 2006;16(5):141–6. 10.1016/j.tcm.2006.03.001 16781946 PMC2749293

[17] Shibata R, Ouchi N, Ito M, Kihara S, Shiojima I, Pimentel DR, et al Adiponectin-mediated modulation of hypertrophic signals in the heart. Nature medicine. 2004;10(12):1384–9. 10.1038/nm1137 15558058 PMC2828675

[18] Fujita K, Maeda N, Sonoda M, Ohashi K, Hibuse T, Nishizawa H, et al Adiponectin protects against angiotensin II-induced cardiac fibrosis through activation of PPAR-alpha. Arteriosclerosis, thrombosis, and vascular biology. 2008;28(5):863–70. 10.1161/ATVBAHA.107.156687 . 18309113

[19] Cao T, Gao Z, Gu L, Chen M, Yang B, Cao K, et al AdipoR1/APPL1 potentiates the protective effects of globular adiponectin on angiotensin II-induced cardiac hypertrophy and fibrosis in neonatal rat atrial myocytes and fibroblasts. PloS one. 2014;9(8):e103793 10.1371/journal.pone.0103793 25099270 PMC4123880

[20] Li H, Yao W, Irwin MG, Wang T, Wang S, Zhang L, et al Adiponectin ameliorates hyperglycemia-induced cardiac hypertrophy and dysfunction by concomitantly activating Nrf2 and Brg1. Free radical biology & medicine. 2015;84:311–21. 10.1016/j.freeradbiomed.2015.03.007 . 25795513

[21] Lee Y, Kim BK, Lim YH, Kim MK, Choi BY, Shin J. The relationship between adiponectin and left ventricular mass index varies with the risk of left ventricular hypertrophy. PloS one. 2013;8(7):e70246 10.1371/journal.pone.0070246 23894624 PMC3722139

[22] Amin RH, Mathews ST, Alli A, Leff T. Endogenously produced adiponectin protects cardiomyocytes from hypertrophy by a PPARgamma-dependent autocrine mechanism. American journal of physiology Heart and circulatory physiology. 2010;299(3):H690–8. 10.1152/ajpheart.01032.2009 20622112 PMC2944479

[23] Koitabashi N, Arai M, Kogure S, Niwano K, Watanabe A, Aoki Y, et al Increased connective tissue growth factor relative to brain natriuretic peptide as a determinant of myocardial fibrosis. Hypertension. 2007;49(5):1120–7. 10.1161/HYPERTENSIONAHA.106.077537 . 17372041

[24] Morikawa H, Tamori A, Nishiguchi S, Enomoto M, Habu D, Kawada N, et al Expression of connective tissue growth factor in the human liver with idiopathic portal hypertension. Molecular medicine. 2007;13(5–6):240–5. 10.2119/2006-00093.Morikawa 17622321 PMC1906684

[25] Rosin NL, Falkenham A, Sopel MJ, Lee TD, Legare JF. Regulation and role of connective tissue growth factor in AngII-induced myocardial fibrosis. The American journal of pathology. 2013;182(3):714–26. 10.1016/j.ajpath.2012.11.014 . 23287510

[26] Leask A. Potential therapeutic targets for cardiac fibrosis: TGFbeta, angiotensin, endothelin, CCN2, and PDGF, partners in fibroblast activation. Circulation research. 2010;106(11):1675–80. 10.1161/CIRCRESAHA.110.217737 . 20538689

[27] Wang B, Haldar SM, Lu Y, Ibrahim OA, Fisch S, Gray S, et al The Kruppel-like factor KLF15 inhibits connective tissue growth factor (CTGF) expression in cardiac fibroblasts. Journal of molecular and cellular cardiology. 2008;45(2):193–7. 10.1016/j.yjmcc.2008.05.005 18586263 PMC2566509

[28] Li G, Xie Q, Shi Y, Li D, Zhang M, Jiang S, et al Inhibition of connective tissue growth factor by siRNA prevents liver fibrosis in rats. The journal of gene medicine. 2006;8(7):889–900. 10.1002/jgm.894 . 16652398

[29] Sriramula S, Haque M, Majid DS, Francis J. Involvement of tumor necrosis factor-alpha in angiotensin II-mediated effects on salt appetite, hypertension, and cardiac hypertrophy. Hypertension. 2008;51(5):1345–51. 10.1161/HYPERTENSIONAHA.107.102152 18391105 PMC2736909

[30] Chen T, Liu J, Li N, Wang S, Liu H, Li J, et al Mouse SIRT3 attenuates hypertrophy-related lipid accumulation in the heart through the deacetylation of LCAD. PloS one. 2015;10(3):e0118909 10.1371/journal.pone.0118909 25748450 PMC4351969

[31] Li CB, Li XX, Chen YG, Zhang C, Zhang MX, Zhao XQ, et al Effects and mechanisms of PPARalpha activator fenofibrate on myocardial remodelling in hypertension. Journal of cellular and molecular medicine. 2009;13(11–12):4444–52. 10.1111/j.1582-4934.2008.00484.x . 18754816 PMC4515060

[32] Li Y, Ma HL, Han L, Liu WY, Zhao BX, Zhang SL, et al Novel ferrocenyl derivatives exert anti-cancer effect in human lung cancer cells in vitro via inducing G1-phase arrest and senescence. Acta pharmacologica Sinica. 2013;34(7):960–8. 10.1038/aps.2013.19 23645009 PMC4002608

[33] Konishi M, Haraguchi G, Ohigashi H, Ishihara T, Saito K, Nakano Y, et al Adiponectin protects against doxorubicin-induced cardiomyopathy by anti-apoptotic effects through AMPK up-regulation. Cardiovascular research. 2011;89(2):309–19. 10.1093/cvr/cvq335 . 20978005

[34] McCollum LT, Gallagher PE, Ann Tallant E. Angiotensin-(1–7) attenuates angiotensin II-induced cardiac remodeling associated with upregulation of dual-specificity phosphatase 1. American journal of physiology Heart and circulatory physiology. 2012;302(3):H801–10. 10.1152/ajpheart.00908.2011 22140049 PMC3353789

[35] Liu YL, Huang CC, Chang CC, Chou CY, Lin SY, Wang IK, et al Hyperphosphate-Induced Myocardial Hypertrophy through the GATA-4/NFAT-3 Signaling Pathway Is Attenuated by ERK Inhibitor Treatment. Cardiorenal medicine. 2015;5(2):79–88. 10.1159/000371454 25999956 PMC4427153

[36] Zhong L, Chiusa M, Cadar AG, Lin A, Samaras S, Davidson JM, et al Targeted inhibition of ANKRD1 disrupts sarcomeric ERK-GATA4 signal transduction and abrogates phenylephrine-induced cardiomyocyte hypertrophy. Cardiovascular research. 2015;106(2):261–71. 10.1093/cvr/cvv108 25770146 PMC4481572

[37] Li L, Zhang ZG, Lei H, Wang C, Wu LP, Wang JY, et al Angiotensin II reduces cardiac AdipoR1 expression through AT1 receptor/ROS/ERK1/2/c-Myc pathway. PloS one. 2013;8(1):e49915 10.1371/journal.pone.0049915 23349663 PMC3551944

[38] Wen P, Song D, Ye H, Wu X, Jiang L, Tang B, et al Circulating MiR-133a as a biomarker predicts cardiac hypertrophy in chronic hemodialysis patients. PloS one. 2014;9(10):e103079 10.1371/journal.pone.0103079 25313674 PMC4196728

[39] Castoldi G, Di Gioia CR, Bombardi C, Catalucci D, Corradi B, Gualazzi MG, et al MiR-133a regulates collagen 1A1: potential role of miR-133a in myocardial fibrosis in angiotensin II-dependent hypertension. Journal of cellular physiology. 2012;227(2):850–6. 10.1002/jcp.22939 . 21769867

[40] Koutsoulidou A, Kyriakides TC, Papadimas GK, Christou Y, Kararizou E, Papanicolaou EZ, et al Elevated Muscle-Specific miRNAs in Serum of Myotonic Dystrophy Patients Relate to Muscle Disease Progress. PloS one. 2015;10(4):e0125341 10.1371/journal.pone.0125341 25915631 PMC4411125

[41] Sang HQ, Jiang ZM, Zhao QP, Xin F. MicroRNA-133a improves the cardiac function and fibrosis through inhibiting Akt in heart failure rats. Biomedicine & pharmacotherapy = Biomedecine & pharmacotherapie. 2015;71:185–9. 10.1016/j.biopha.2015.02.030 . 25960234

[42] Drawnel FM, Wachten D, Molkentin JD, Maillet M, Aronsen JM, Swift F, et al Mutual antagonism between IP(3)RII and miRNA-133a regulates calcium signals and cardiac hypertrophy. The Journal of cell biology. 2012;199(5):783–98. 10.1083/jcb.201111095 23166348 PMC3514786

[43] Myers R, Timofeyev V, Li N, Kim C, Ledford HA, Sirish P, et al Feedback Mechanisms for Cardiac-Specific MicroRNAs and cAMP Signaling in Electrical Remodeling. Circulation Arrhythmia and electrophysiology. 2015;8(4):942–50. 10.1161/CIRCEP.114.002162 25995211 PMC4545299

[44] Cheng Y, Ji R, Yue J, Yang J, Liu X, Chen H, et al MicroRNAs are aberrantly expressed in hypertrophic heart: do they play a role in cardiac hypertrophy? The American journal of pathology. 2007;170(6):1831–40. 10.2353/ajpath.2007.061170 17525252 PMC1899438

[45] Zhao Y, Samal E, Srivastava D. Serum response factor regulates a muscle-specific microRNA that targets Hand2 during cardiogenesis. Nature. 2005;436(7048):214–20. 10.1038/nature03817 . 15951802

[46] Liu N, Williams AH, Kim Y, McAnally J, Bezprozvannaya S, Sutherland LB, et al An intragenic MEF2-dependent enhancer directs muscle-specific expression of microRNAs 1 and 133. Proceedings of the National Academy of Sciences of the United States of America. 2007;104(52):20844–9. 10.1073/pnas.0710558105 18093911 PMC2409229

[47] Tomas E, Tsao TS, Saha AK, Murrey HE, Zhang Cc C, Itani SI, et al Enhanced muscle fat oxidation and glucose transport by ACRP30 globular domain: acetyl-CoA carboxylase inhibition and AMP-activated protein kinase activation. Proceedings of the National Academy of Sciences of the United States of America. 2002;99(25):16309–13. 10.1073/pnas.222657499 12456889 PMC138607

[48] Wu X, Motoshima H, Mahadev K, Stalker TJ, Scalia R, Goldstein BJ. Involvement of AMP-activated protein kinase in glucose uptake stimulated by the globular domain of adiponectin in primary rat adipocytes. Diabetes. 2003;52(6):1355–63. . 12765944 10.2337/diabetes.52.6.1355

[49] Yamauchi T, Kamon J, Minokoshi Y, Ito Y, Waki H, Uchida S, et al Adiponectin stimulates glucose utilization and fatty-acid oxidation by activating AMP-activated protein kinase. Nature medicine. 2002;8(11):1288–95. 10.1038/nm788 . 12368907

[50] Ouchi N, Kobayashi H, Kihara S, Kumada M, Sato K, Inoue T, et al Adiponectin stimulates angiogenesis by promoting cross-talk between AMP-activated protein kinase and Akt signaling in endothelial cells. The Journal of biological chemistry. 2004;279(2):1304–9. 10.1074/jbc.M310389200 14557259 PMC4374490

[51] Matkovich SJ, Wang W, Tu Y, Eschenbacher WH, Dorn LE, Condorelli G, et al MicroRNA-133a protects against myocardial fibrosis and modulates electrical repolarization without affecting hypertrophy in pressure-overloaded adult hearts. Circulation research. 2010;106(1):166–75. 10.1161/CIRCRESAHA.109.202176 19893015 PMC2804031

