# Supplementary material for: Adiponectin Upregulates MiR-133a in Cardiac Hypertrophy through AMPK Activation and Reduced ERK1/2 Phosphorylation
Source: PLoS One. 2016 Feb 4;11(2):e0148482. doi: 10.1371/journal.pone.0148482 (PMC4741527; doi:10.1371/journal.pone.0148482)
Supplement: S1 File — The left ventricular end-diastolic posterior wall thickness (LVPWd) (Fig A), and end-diastolic interventricular septal thickness (IVSd) (Fig B) and Left ventricular weight index (LVW/BW) were increased induced by Ang II (Fig C). ANF (Fig D) and (Fig E) BNP mRNA level was elevated by Ang II. The mRNA expression was calculated as fold induction compared to the control 7d group Plasma APN was decreased by Ang II infusion (Fig F). (n = 6 for each group. *, p < 0.05 vs control. **, p < 0.01 vs control). (DOCX) [file pone.0148482.s001.docx]

**
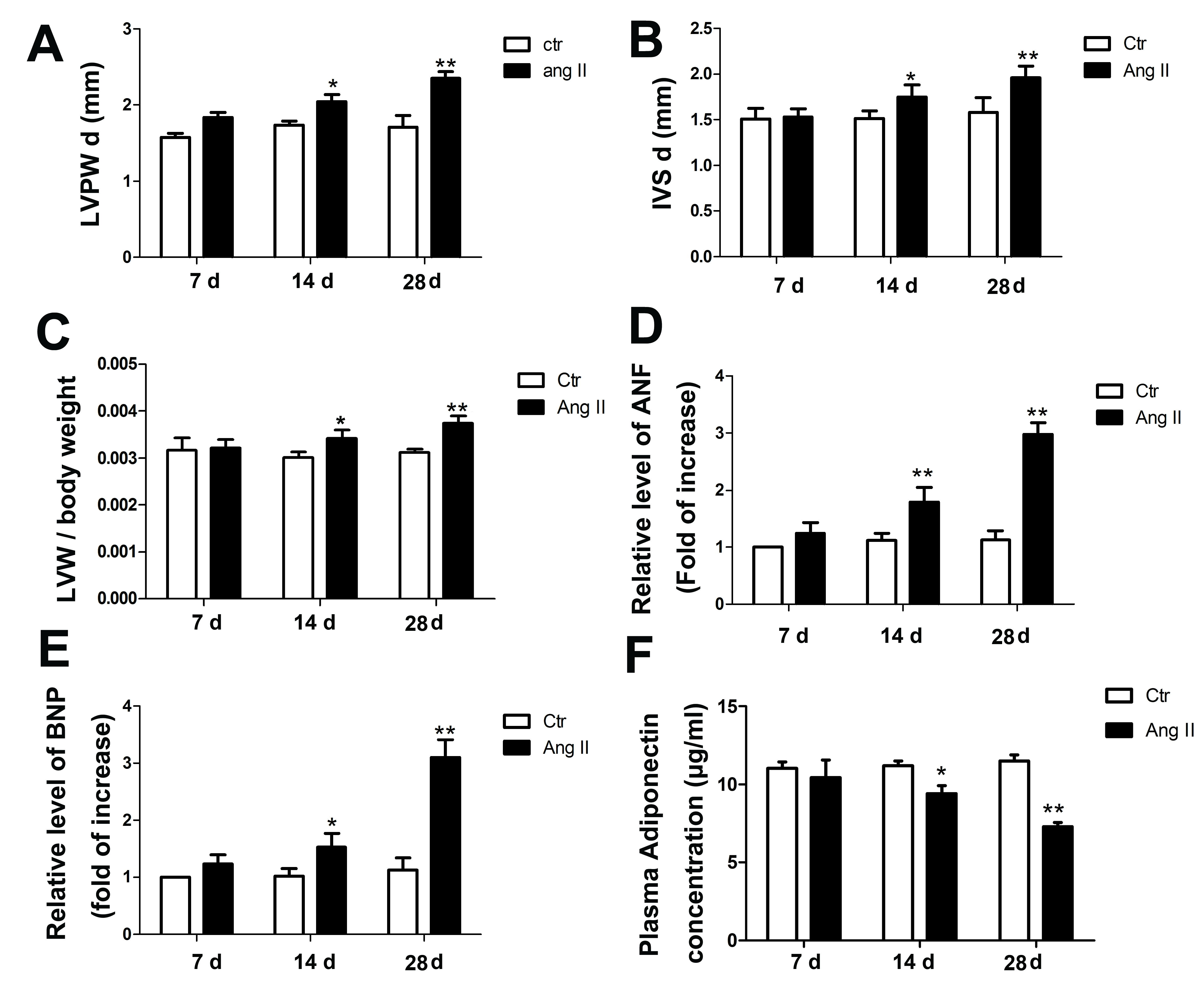
**

**S1 File. Continuous Ang-II infusion results in cardiac hypertrophy.** The left ventricular end-diastolic posterior wall thickness (LVPWd) (**Figure A**) and end-diastolic interventricular septal thickness (IVSd) (**Figure B**) and Left ventricular weight index (LVW/BW) were increased induced by Ang II (**Figure C**). ANF (**Figure D**) and BNP (**Figure E**) mRNA level was elevated by Ang II. The mRNA expression was calculated as fold induction compared to the control 7 d group. Plasma APN was decreased by Ang II infusion (**Figure F**). (n = 6 for each group. *, *p* < 0.05 vs control. **, *p* < 0.01 vs control).
