# Supplementary material for: Adiponectin Upregulates MiR-133a in Cardiac Hypertrophy through AMPK Activation and Reduced ERK1/2 Phosphorylation
Source: PLoS One. 2016 Feb 4;11(2):e0148482. doi: 10.1371/journal.pone.0148482 (PMC4741527; doi:10.1371/journal.pone.0148482)
Supplement: S2 File — (**, p < 0.01, n = 6 for each group). (DOCX) [file pone.0148482.s002.docx]

**
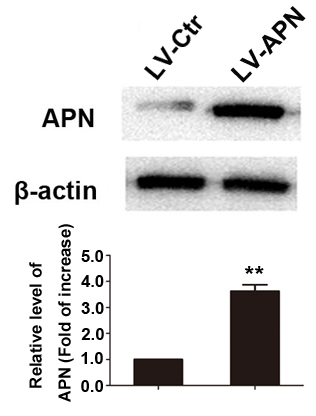
**

**S2 File. Lentiviral vector-mediated APN overexpression was determined by western blot.** (**, *p* < 0.01, n = 6 for each group).
