# Supplementary material for: Adiponectin Upregulates MiR-133a in Cardiac Hypertrophy through AMPK Activation and Reduced ERK1/2 Phosphorylation
Source: PLoS One. 2016 Feb 4;11(2):e0148482. doi: 10.1371/journal.pone.0148482 (PMC4741527; doi:10.1371/journal.pone.0148482)
Supplement: S3 File — qRT-PCR was performed to detect miR-133a level under different treatment (**, p < 0.01 vs control). (DOCX) [file pone.0148482.s003.docx]

**
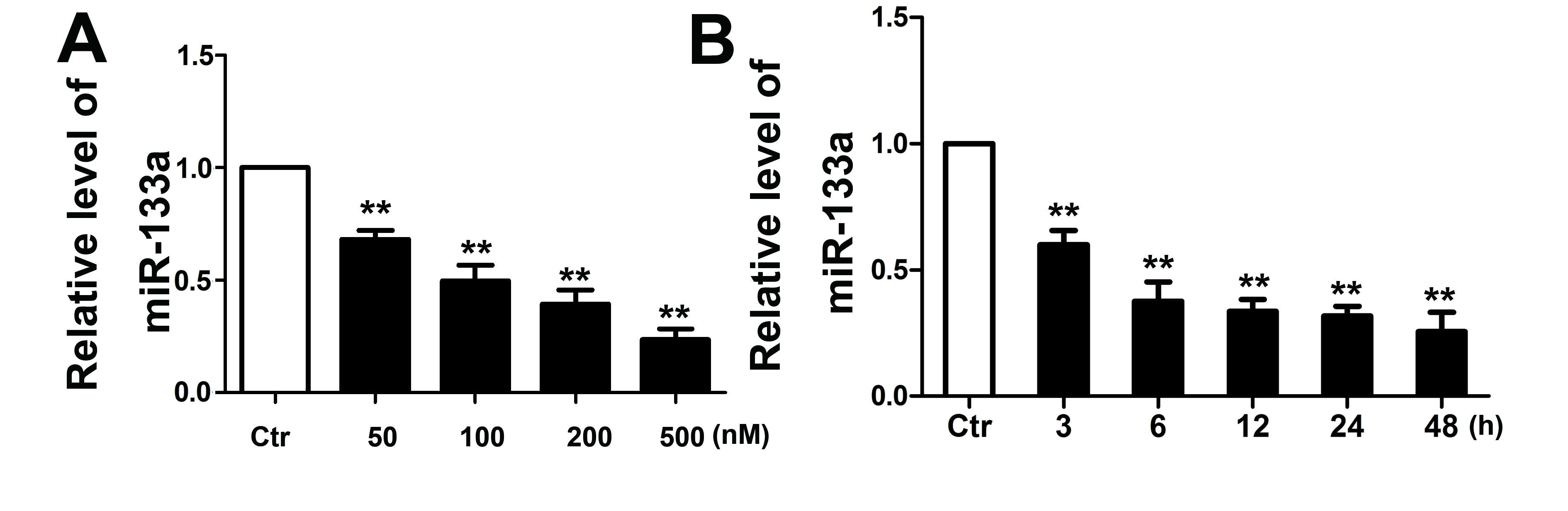
**

**S3 File. Ang II downregulate miR-133a level in a dose and time- dependent manner.** qRT-PCR was performed to detect miR-133a level under different treatment (**, *p* < 0.01 vs control).
