# Supplementary material for: Adiponectin Upregulates MiR-133a in Cardiac Hypertrophy through AMPK Activation and Reduced ERK1/2 Phosphorylation
Source: PLoS One. 2016 Feb 4;11(2):e0148482. doi: 10.1371/journal.pone.0148482 (PMC4741527; doi:10.1371/journal.pone.0148482)
Supplement: S4 File — (**, p < 0.01). (DOCX) [file pone.0148482.s004.docx]

**
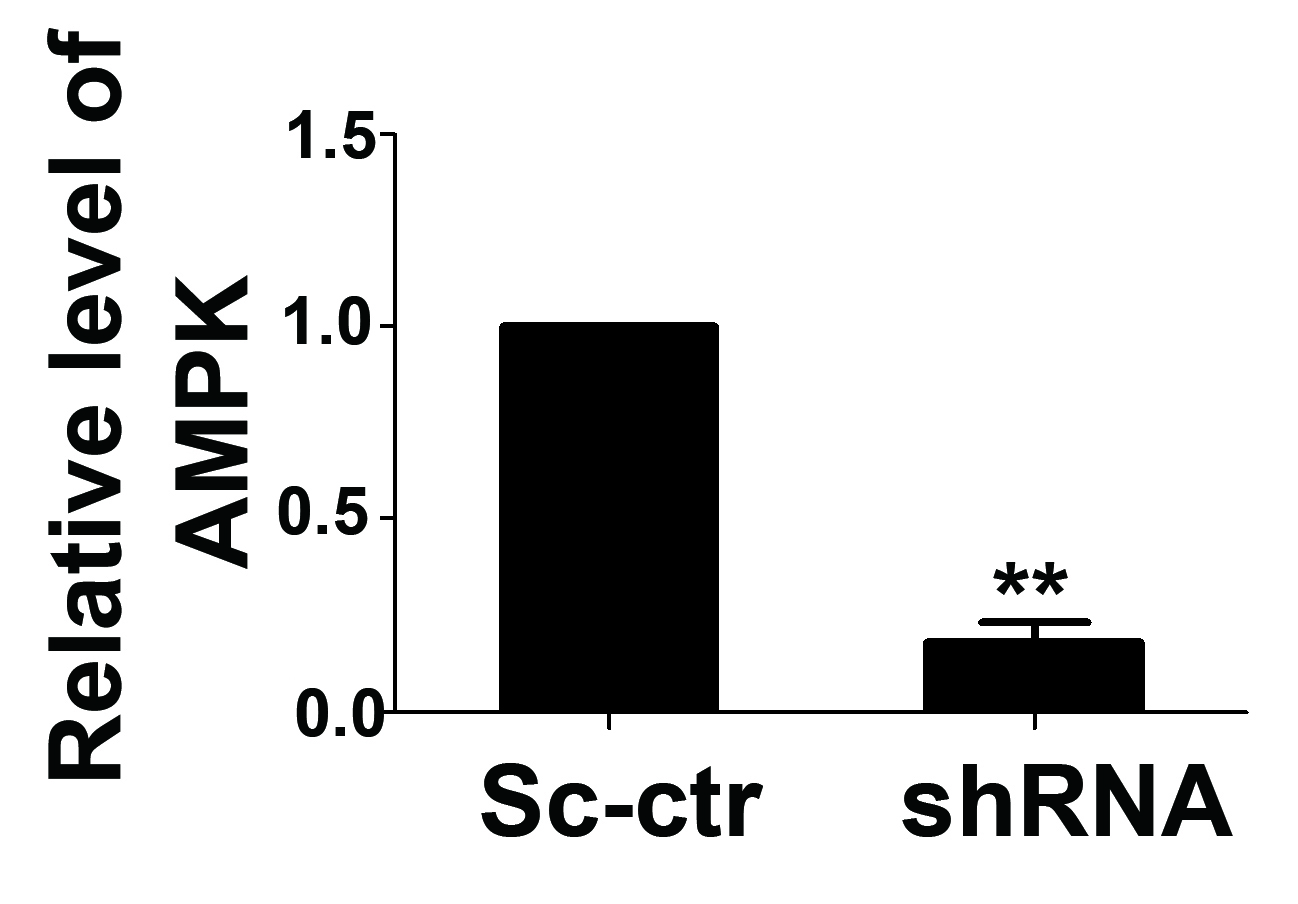
**

**S4 File. qPCR was performed to examine AMPK mRNA level after transfected with lentiviral AMPK shRNA.** (**, *p* < 0.01 vs Sc-ctr).
