# Supplementary material for: Adiponectin Upregulates MiR-133a in Cardiac Hypertrophy through AMPK Activation and Reduced ERK1/2 Phosphorylation
Source: PLoS One. 2016 Feb 4;11(2):e0148482. doi: 10.1371/journal.pone.0148482 (PMC4741527; doi:10.1371/journal.pone.0148482)
Supplement: S5 File — (**, p < 0.01 vs control. *, p < 0.05 vs control). (DOCX) [file pone.0148482.s005.docx]

**
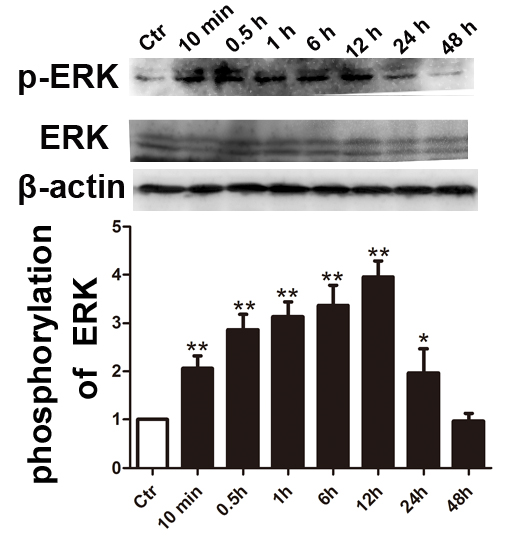
**

**S5 File. Western blot was performed to determine phosphorylation of ERK in NRVMs treated with Ang II for different time.** (**, *p* < 0.01 vs control. *, *p* < 0.05 vs control).
