# Supplementary figures and images for: Adiponectin Upregulates MiR-133a in Cardiac Hypertrophy through AMPK Activation and Reduced ERK1/2 Phosphorylation
Source: PLoS One. 2016 Feb 4;11(2):e0148482. doi: 10.1371/journal.pone.0148482 (PMC4741527; doi:10.1371/journal.pone.0148482)

**
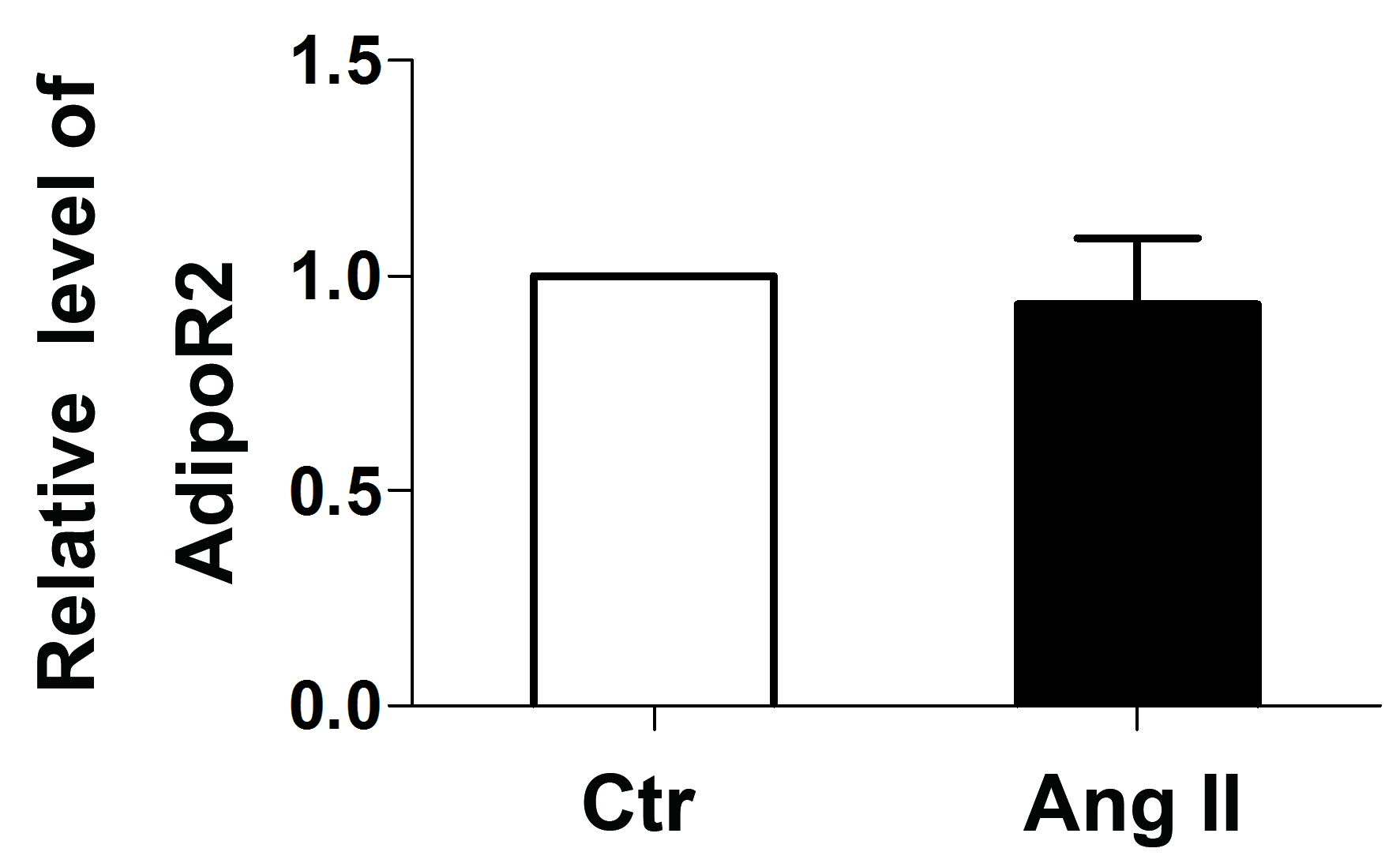
**

**S7 File. AdipoR2 mRNA level was not changed by stimulation with Ang II.**

Supplement: S7 File — (DOCX) [file pone.0148482.s007.docx]
